# Characteristics of Genetic Variations Associated With Lennox-Gastaut Syndrome in Korean Families

**DOI:** 10.3389/fgene.2020.590924

**Published:** 2021-01-20

**Authors:** Jin Ok Yang, Min-Hyuk Choi, Ji-Yong Yoon, Jeong-Ju Lee, Sang Ook Nam, Soo Young Jun, Hyeok Hee Kwon, Sohyun Yun, Su-Jin Jeon, Iksu Byeon, Debasish Halder, Juhyun Kong, Byungwook Lee, Jeehun Lee, Joon-Won Kang, Nam-Soon Kim

**Affiliations:** ^1^Korea BioInformation Center, Korea Research Institute of Bioscience and Biotechnology, Daejeon, South Korea; ^2^Department of Bio and Brain Engineering, Korea Advanced Institute of Science and Technology, Daejeon, South Korea; ^3^Rare-Disease Research Center, Korea Research Institute of Bioscience and Biotechnology, Daejeon, South Korea; ^4^Department of Functional Genomics, Korea University of Science and Technology, Daejeon, South Korea; ^5^Department of Pediatrics, Pusan National University Children’s Hospital, Pusan National University School of Medicine, Yangsan, South Korea; ^6^Department of Medical Science and Anatomy, Chungnam National University, Daejeon, South Korea; ^7^Department of Pediatrics, Samsung Medical Center, Sungkyunkwan University School of Medicine, Seoul, South Korea; ^8^Department of Pediatrics and Medical Science, Chungnam National University Hospital, College of Medicine, Chungnam National University, Daejeon, South Korea

**Keywords:** Lennox-Gastaut syndrome, epilepsy, whole-exome sequencing, genetic variation, Rare-diseases

## Abstract

Lennox-Gastaut syndrome (LGS) is a severe type of childhood-onset epilepsy characterized by multiple types of seizures, specific discharges on electroencephalography, and intellectual disability. Most patients with LGS do not respond well to drug treatment and show poor long-term prognosis. Approximately 30% of patients without brain abnormalities have unidentifiable causes. Therefore, accurate diagnosis and treatment of LGS remain challenging. To identify causative mutations of LGS, we analyzed the whole-exome sequencing data of 17 unrelated Korean families, including patients with LGS and LGS-like epilepsy without brain abnormalities, using the Genome Analysis Toolkit. We identified 14 mutations in 14 genes as causes of LGS or LGS-like epilepsy. 64 percent of the identified genes were reported as LGS or epilepsy-related genes. Many of these variations were novel and considered as pathogenic or likely pathogenic. Network analysis was performed to classify the identified genes into two network clusters: neuronal signal transmission or neuronal development. Additionally, knockdown of two candidate genes with insufficient evidence of neuronal functions, *SLC25A39* and *TBC1D8*, decreased neurite outgrowth and the expression level of *MAP2*, a neuronal marker. These results expand the spectrum of genetic variations and may aid the diagnosis and management of individuals with LGS.

## Introduction

Lennox-Gastaut syndrome (LGS) is a severe form of childhood-onset epilepsy with a heterogeneous etiology, and epileptiform abnormalities may contribute to progressive dysfunction (Lund et al., 2014; Asadi-Pooya, 2018). The primary features of LGS are multiple types of seizures (generalized tonic, atonic, and atypical absence), generalized slow spike-and-wave or generalized paroxysmal fast activity discharges on electroencephalography, and intellectual disabilities (Camfield, 2011; Asadi-Pooya, 2018). In approximately 70% of patients with LGS, this disease is caused by brain damage, infection, and brain malformation. Thirty percent of LGS patients do not present abnormalities in brain imaging, and thus the cause of their condition is unclear (Asadi-Pooya, 2018). Functional magnetic resonance imaging studies showed that abnormal network connectivity in subcortical structures causes LGS (Pedersen et al., 2015). Hence, several researchers have focused on screening genetic risk factors from patients with LGS without abnormalities by next-generation sequencing.

Recently, several causative genetic variations related to LGS and epilepsy, which play important roles in the development of these syndromes, have been detected by whole-exome sequencing (WES; Allen et al., 2013, 2015; Lund et al., 2013; Terrone et al., 2014; Zerem et al., 2016; Wang et al., 2017; Asadi-Pooya, 2018; Dunn et al., 2018; Tumiene et al., 2018). Nevertheless, effective treatments and an understanding of the genetic basis of LGS are lacking because the biological mechanisms of LGS are not well-understood. The seizure frequency is either controlled by administering anti-epileptic medicine to patients or stimulating the vagus nerve. Therefore, comprehensive information from LGS-related genetic variations and networks is required to identify markers influencing LGS.

In this study, we examined novel candidate genetic variations and networks associated with LGS without brain abnormalities as genetic markers. First, we collected 58 WES datasets from 17 Korean families with a clinical history of LGS or LGS-like epilepsy without brain abnormalities. We investigated the causative variations in each family and relationships among genes and these variations, and then estimated the genetic risk factors for LGS. We found that these variations contained 14 mutations, including *de novo*, autosomal recessive (AR), and X-linked mutations. Several genes showed novel variations and were found to be associated with LGS or epilepsy. Depletion of two candidate genes with insufficient evidence of neuronal functions decreased neurite outgrowth in the SH-SY5Y cell line. This finding provides a more informative resource for LGS-related genetic variations and may contribute to the diagnosis of and therapeutic platform development for LGS and LGS-like epilepsy.

## Materials and Methods

### Clinical Specimens of Korean Patients With LGS

We collected 58 samples from 17 affected individuals and 41 unaffected individuals. These subjects were from 17 unrelated Korean families containing either 10 patients with LGS or 7 patients with LGS-like epilepsy (Figure 2). Patients with an incomplete phenotype of LGS were categorized as having LGS-like epilepsy. The magnetic resonance imaging results were normal (15 patients) or showed non-epileptogenic abnormalities (2 patients), including arachnoid cyst, posterior fossa, and brain atrophy. The mean age at seizure onset was 3.3 years (range 0.16–12 years). All patients diagnosed with LGS or LGS-like epilepsy were older than 3 years. Affected individuals presented with various seizure types: tonic (16/17), atonic (6/17), generalized tonic-clonic (4/17), myoclonic (4/17), and atypical absence (8/17; Table 1). All patients underwent neurologic and genetic evaluations based on the clinical criteria of LGS by an experienced neurologist (Camfield, 2011). LGS is characterized by: (1) a clinical triad of various types of generalized seizures, including generalized tonic, atonic, myoclonic, atypical absence seizures, and epileptic spasms; (2) generalized slow spikes and waves and/or generalized paroxysmal rapid activity for electroencephalography; and (3) progressive developmental regression after seizure onset. LGS-like epilepsy is broadly defined to result in at least two types of generalized seizures, including tonic seizures, or a combination of atonic and atypical absence seizures, learning disabilities, resistance to treatment, and bilateral synchronous epileptic discharges. Ethical approval for the study was obtained from the Institutional Review Board and Ethics Committee at the Chungnam National University Hospitals and Korea Research Institute of Bioscience and Biotechnology. Written informed consent was obtained from all participants or their legal representatives. Available clinical information on these patients is shown in Table 1.

**TABLE 1:** Clinical features of patients with LGS and LGS-like epilepsy.

Family/Patient number (Gender)	Seizure onset	Seizure types	AEDs	Response to therapy	EEG	Brain MRI	Epileptic syndrome	Additional symptom	Phenotypes in unaffected individuals
1 (M)	9 y	Tonic, atonic, atypical absence	Valproic acid, Rufinamide	Intractable	GPFA, GSSW	Normal	LGS	DD	None
2 (F)	4 m	Tonic, atypical absence	Valproic acid, Clobazam, Rufinamide, Levetiracetam	Intractable	GPFA, GSSW	Normal	LGS	DD	None
3 (M)	12 y	Tonic, atonic	Valproic acid, Lamotrigine, Rufinamide, Levetiracetam, Lacosamide	Intractable	GPFA, GSSW	Normal	LGS	DD	None
4 (F)	2 y	Tonic, atypical absence	Valproic acid, Rufinamide	Intractable	GPFA, GSSW	Normal	LGS	DD	None
5 (M)	7 y	Tonic, atonic	Rufinamide, Valproic acid, Oxcarbazepine, Topiramate, Levetiracetam, Clobazam	Intractable	GPFA, GSSW	Normal	LGS	DD	None
6 (M)	1 y	Tonic, atonic, atypical absence	Valproic acid, Levetiracetam, Zonisamide, Clobazam	Intractable	GPFA, GSSW	Normal	LGS	DD	Mild ID (Maternal grandmother, mother)
7 (M)	4 m	Tonic, myoclonic	Topiramate, Valproic acid, Clobazam, Lamotrigine, Perampanel	Intractable	GPFA, GSSW	Normal	LGS	Infantile spasms	None
8 (M)	6 m	Tonic, atypical absence	Clobazam, Vigabatrin, Rufinamide, Levetiracetam	Intractable	GPFA, GSSW	Arachnoid cyst, posterior fossa	LGS	Infantile spasms	None
9 (M)	19 m	Generalized tonic-clonic, tonic, atypical absence, myoclonic	Vigabatrin, Clobazam, Zonisamide	Intractable	GPFA, GSSW	Normal	LGS	DD, ID	None
10 (M)	7 m	Generalized tonic-clonic, tonic, myoclonic	Valproic acid, Phenobarbital, Lacosamide, Oxcarbazepine, Rufinamide	Intractable	GPFA, GSSW	Normal	LGS	DD, ID	None
11 (F)	4 y	Tonic, atonic	Valproic acid	Good	GSSW	Normal	LGS-like	DD	None
12 (M)	2 y	Tonic	Valproic acid, Clobazam, Rufinamide	Good	GPFA, GSSW	Normal	LGS-like	DD	None
13 (M)	3 y	Tonic, atonic	Levetiracetam	Good	GSSW	Normal	LGS-like	DD	None
14 (M)	3 y	Tonic, atypical absence	Valproic acid	Intractable	GSSW	Normal	LGS-like	ID	None
15 (F)	3 m	Tonic, myoclonic, partial	Topiramate, Valproic acid, Clobazam, Levetiracetam, Vigabatrin, Zonisamide	Intractable	GPFA	Normal	LGS-like	DD, ID	None
16 (M)	10 y	Generalized tonic-clonic, atypical absence	Topiramate, Lamotrigine, Valproic acid, Clobazam, Perampanel	Intractable	GSSW	Normal	LGS-like	DD, ID	None
17 (M)	2 m	Generalized tonic-clonic, tonic	Topiramate, Valproic acid, Clobazam, Vigabatrin, Levetiracetam, Phenobarbital	Intractable	GPFA	Brain atrophy	LGS-like	DD, ID	None

*Abbreviations: y, year; m, month; AEDs, Anti-epileptic drugs; EEG, Electroencephalography; GPFA, Generalized paroxysmal fast activity; GSSW, Generalized slow spike-and-wave; DD, Developmental delay; ID, Intellectual disability; and MRI, Magnetic resonance imaging.*

### Analysis of Genetic Variations and Annotation

**FIGURE 1:**
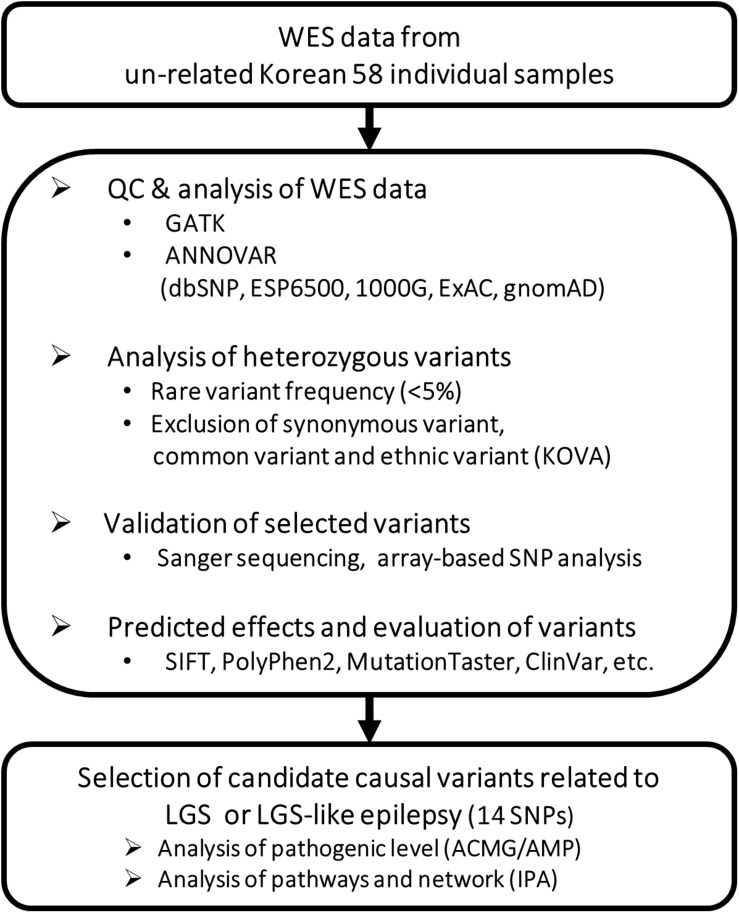
Workflow for identifying potential causative genetic variants related to LGS or LGS-like epilepsy.

We obtained high-throughput WES data from all participants by HiSeq 2500 (Illumina, San Diego, CA, United States). All sequencing reads were mapped to the human reference genome GRCh38/hg38 by using Burrows-Wheeler Aligner software (v0.7.17). Variant calling and functional annotation were performed using the Genome Analysis Toolkit (GATK4, Broad Institute, MA, United States) and ANNOVAR (Version 2018Apr16), respectively, (McKenna et al., 2010; Wang et al., 2010). For the rare-disease study, mutations with less than 5% minor allele frequency in our data were selected using genetic variation data in the Single Nucleotide Polymorphism Database (dbSNP, https://www.ncbi.nlm.nih.gov/snp/), NHLBI Grand Opportunity Exome Sequencing Project database (ESP6500, https://evs.gs.washington.edu/EVS/), and 1000 Genomes Project^1^. We also examined the allele frequency of candidate genetic variations using the Exome Aggregation Consortium (ExAC, http://exac.broadinstitute.org/) and Genome Aggregation Database (gnomAD, https://gnomad.broadinstitute.org/), and ethnic mutations were removed by referencing 1,055 healthy Korean WES data (KOVA v1, http://kobic.re.kr/kova/). To validate these genetic variants, we carried out Sanger sequencing using an ABI3730XL DNA sequencer (Applied Biosystems, Foster City, CA, United States). We also identified copy number variations (CNVs) by CODEX, which is based on a multi-sample normalization model (Jiang et al., 2015). It includes terms that specifically remove biases due to GC content, exon length, and targeting and amplification efficiency. We normalized the WES read count data of each patient with LGS and LGS-like epilepsy based on the read depth of unaffected individuals, after which we derived *de novo* CNVs. CNVs were validated by array-based SNP analysis (Illumina Infinium Omni2.5-8 BeadChip; Supplementary Methods). The family dataset without candidate markers was excluded. The summarized workflow is shown in Figure 1.

**FIGURE 2:**
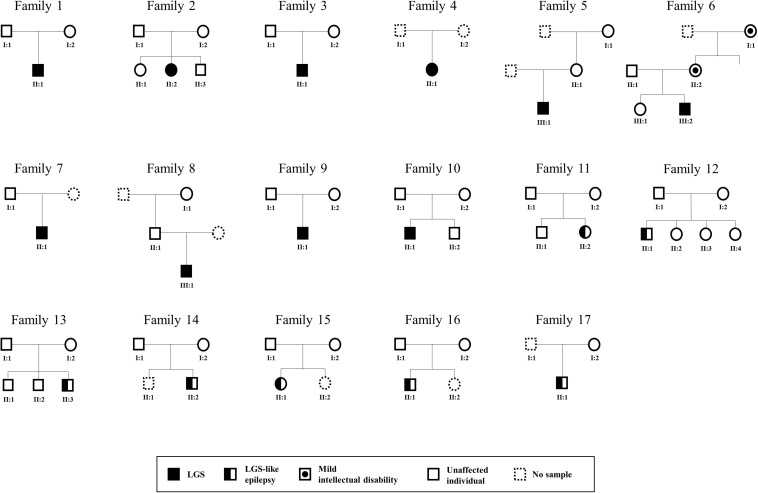
Pedigree of LGS or LGS-like epilepsy families. Males and females are represented as squares and circles, respectively. Patients with LGS are indicated as completely filled symbols, and those with LGS-like epilepsy are indicated as half-filled symbols. The circle with dots indicates females with mild intellectual disabilities.

### Pathogenicity Prediction and Pathway Analysis

We investigated the pathogenicity of all mutations using the ClinVar database and prediction algorithms in ANNOVAR (PolyPhen2, SIFT, LRT, and MutationTaster; Chun and Fay, 2009; Adzhubei et al., 2010; Schwarz et al., 2010; Sim et al., 2012). Additionally, we evaluated pathogenicity using criteria described in the American College of Medical Genetics and Association for Molecular Pathology guidelines, after which we selected genes with damaging, possibly damaging, pathogenic, or likely pathogenic mutations as candidate genes of LGS and LGS-like epilepsy (Richards et al., 2015).

We performed Ingenuity Pathway Analysis (QIAGEN, Hilden, Germany) to explore the pathways and networks associated with selected candidate gene sets, and classified genes according to their functions by using Database for Annotation, Visualization and Integrated Discovery (DAVID, https://david.ncifcrf.gov).

### Neurite Outgrowth Assay

Human SH-SY5Y neuroblastoma cells were transfected with target siRNAs for 24 h and then treated with retinoic acid for 48 h. Cells were then fixed in 4% paraformaldehyde for 1 h at room temperature (RT), permeabilized with 0.1% Triton X-100 in PBS for 15 min, and incubated in blocking reagent (5% normal fetal bovine serum in PBS) for 1 h. Cells were incubated with primary antibody against MAP2, a neuronal marker (Mouse Anti-MAP2, Abcam, Cambridge, United Kingdom), at 4°C overnight, followed by incubation with a secondary antibody (Alexa Fluor 488, Goat anti-mouse, Life Technologies, California, United States) at RT for 1 h. For nuclear counterstaining, the cells were incubated with DAPI solution (300 nM in PBS) for 5 min at RT and then observed with a fluorescence microscope (Eclipse Ti-S, Nikon, Tokyo, Japan). To measure neurite length in SH-SY5Y cells, neurite length was calculated as the longest neurite distance from the cell body (direct path to the soma) on each neuron showing MAP2 (green) using ImageJ software (10 calculated cells per group). The mRNA expression levels of target genes and MAP2 were analyzed by real-time PCR.

## Results

### Annotation of Genetic Variations as Candidates for LGS and LGS-Like Epilepsy

We identified 14 mutations as candidates for causing LGS and LGS-like epilepsy. Two mutations were in the splicing site and 12 mutations were in the coding region. Among the coding region mutations, there was one frameshift deletion, one non-frameshift insertion, and 10 single-nucleotide variations (Table 2 and Supplementary Figure 1).

**TABLE 2:** Potential causative genetic variations in patients with LGS or LGS-like epilepsy.

Patient	Inheritance	Gene name	NT, AA change	dbSNP ID	Description	ACMG/AMP	
	
						Criteria	Classification
1	*De novo*	*SLC25A39*	c.C112T, p.R38C	rs757102633	Neuron-related	PM2, PP3	Uncertain significance
3	*De novo*	*TBC1D8*	c.T1547G, p.L516R	None	Neuron-related	PM2, PP3	Uncertain significance
4	Unknown	*SYN1*	c.C1666T, p.R556C	rs1441575488	Epilepsy-related	PM1, PM2, PM6, PP3	Likely pathogenic
5	Unknown	*SHANK3*	c.C3746T, p.P1249L	rs757572910	Epilepsy-related	PM6, PP3	Uncertain significance
6	X-linked	*IQSEC2*	c.G1048A, p.A350T	None	LGS-related	PM1,PM2,PM5, PP3	Likely pathogenic
7	Unknown	*SYN2*	c.A1379G, p.Q460R	rs2289706	Epilepsy-related	PM2, PM6, PP3	Uncertain significance
8	Unknown	*MAGI1*	c.T2475G, p.F825L	None	Neuron-related	PM2, PP3	Uncertain significance
9	AR	*CACNA1A*	c.6975_ 6976ins CAGCAGCAGCAG, p.Q2325_ A2326ins QQQQ	None	LGS- related	PM3	Uncertain significance
10	AR	*FRRS1L*	c.G615T^#^, p.M205I	rs750750976	Epilepsy-related	PVS1, PM1, PM2, PM3, PP3	Pathogenic
11	AR	*SSPO*	c.12608delC, p.Q4204Rfs*41	rs11353848	Neuron-related	PM3, PM4	Uncertain significance
12	*De novo*	*CHD2*	c.443 + 1G > A	None	LGS-related	PVS1, PS2, PM2, PP3	Pathogenic
14	*De novo*	*NRG2*	c.C835T, p.R279C	rs1226659673	Neuron-related	PM2, PP3	Uncertain significance
15	*De novo*	*DNAJC5*	c.C141A, p.N47K	None	Epilepsy-related	PS2, PM2, PP3	Likely pathogenic
17	Unknown	*SCN10A*	c.389 + 2T > C	None	LGS-related	PVS1,PM2,PP3	Pathogenic

*^#^This variation exists in the last nucleotide of exon 3 and is predicted to affect splicing. Abbreviations: AR, Autosomal recessive; NT, Nucleotide; AA, Amino acid; NA, Not available; and Unknown indicates potentially *de novo.**

**FIGURE 3:**
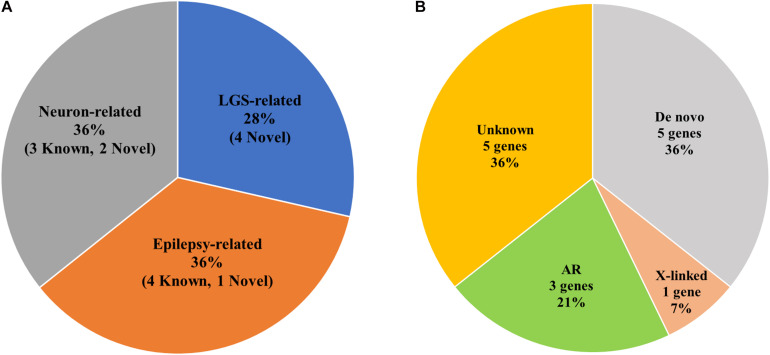
Statistics of genetic variations in candidate genes of LGS or LGS-like epilepsy. **(A)** Pie chart of the frequency of LGS, epilepsy, or neuron-related genes. () indicates the number of genetic variations in candidate genes. **(B)** Ratio of Mendelian inheritance patterns of candidate genes.

*CACNA1A*, *CHD2*, *IQSEC2*, and *SCN10A* were LGS-related genes. *DNAJC5*, *FRRS1L*, *SHANK3*, *SYN1*, and *SYN2* were epilepsy-related genes. *MAGI1*, *NRG2*, *SLC25A39*, *SSPO*, and *TBC1D8* were functionally related to neurons or brain-expressed ion channels (Figure 3A and Table 2). Except for mutations in *FRRS1L*, *NRG2*, *SHANK3*, *SLC25A39*, *SSPO*, *SYN1*, and *SYN2*, the remaining mutations were novel.

### Mendelian Inheritance Pattern

Among the 17 families, one family contained members with mild intellectual disability, whereas disorders were not present in members of the other 16 families (Figure 2 and Table 1). We identified three AR (21%), one X-linked inherited (7%), five *de novo* (36%), and five potentially *de novo* mutations (36%; Figure 3B). Remarkably, as shown in Table 2, mutations in *CACNA1A*, *FRRS1L*, and *SSPO* were detected as AR in families 9, 10, and 11, in which unaffected parents possessed heterozygous mutations and the patients had homozygous mutations. The *IQSEC2* mutation was inherited as X-linked in family 6, in which the maternal grandmother and mother of the patient had mild intellectual disability with heterozygous mutations, whereas the male patient had hemizygous mutations. Genes such as *CHD2*, *DNAJC5*, *NRG2*, *SLC25A39*, and *TBC1D8* showed *de novo* mutations. *MAGI1*, *SCN10A*, *SHANK3*, *SYN1*, and *SYN2* were considered as unknown, potentially *de novo* mutations, as they were found in patients from single-parent or adoptive families (Figure 3B).

### Pathogenicity Validation for Genetic Variations

Mutations in *FRRS1L*, *NRG2*, *SHANK3*, *SLC25A39*, *SSPO*, *SYN1*, and *SYN2* have been reported previously but their functional significance was not determined by ClinVar. In addition, other mutations were not reported in databases such as dbSNP, ClinVar, ExAC, ESP6500, gnomAD, and the 1000 Genomes Project. According to PolyPhen2, SIFT, LRT, and MutationTaster, damaging mutations were found in *DNAJC5*, *FRRS1L*, and *IQSEC2* in all pathogenicity prediction results, with the remaining mutations classified as possibly damaging or of unknown pathogenicity. According to the American College of Medical Genetics/Association for Molecular Pathology guidelines, the *CHD2*, *DNAJC5*, *FRRS1L*, *IQSEC2*, *SCN10A*, and *SYN1* mutations were sorted into pathogenic or likely pathogenic groups. Particularly, the *FRRS1L* mutation (c.615G > *T*, p.205M > *I*) at the last nucleotide of exon 3 may affect splicing. *FRRS1L* is related to epileptic encephalopathy, and its pathogenic variants have been reported previously (Madeo et al., 2016; Han et al., 2017). Based on these findings, the *FRRS1L* mutation was assigned to PVS1. The detailed criteria and classification results are presented in Table 2 and Supplementary Table 1.

### Systematic Analysis of Candidate Genes

**FIGURE 4:**
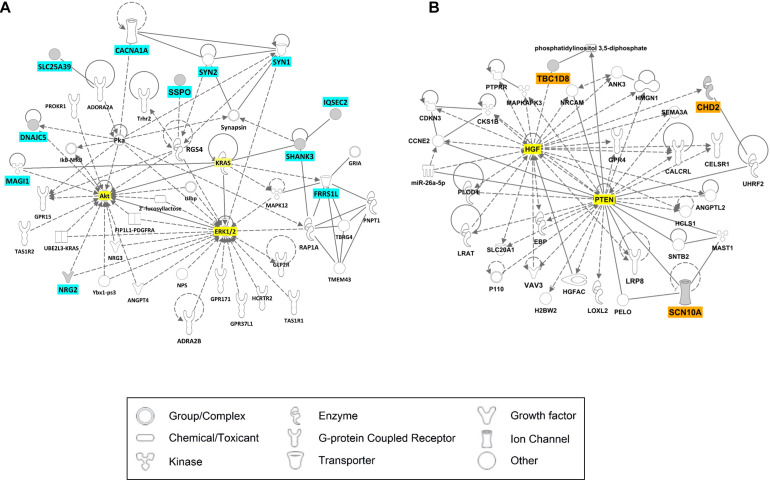
Ingenuity Pathway Analysis (IPA) results for candidate genes of LGS or LGS-like epilepsy. The genes with cyan or orange colors indicate candidate genes selected in this study. The genes with yellow color represent those most relevant to the candidate genes. **(A)** Cell signaling network. **(B)** Neurological disease network.

We examined gene interaction networks and functional annotations to comprehensively understand the relationships among the candidate genes of LGS and LGS-like epilepsy. Based on gene ontology annotations by DAVID, these gene sets were enriched in biological functions including synaptic transmission, ion transport, MAPK cascade, and transcription (Table 3). Ingenuity Pathway Analysis revealed that the candidate genes were divided into two clusters. The first cluster comprised 11 genes (*CACNA1A*, *DNAJC5*, *FRRS1L*, *IQSEC2*, *MAGI1*, *NRG2*, *SHANK3*, *SLC25A39*, *SSPO, SYN1*, and *SYN2*), which functionally belong to synaptogenesis signaling, calcium signaling, and AMPA signaling. This gene set was associated with neurotransmission (Figure 4A). Another cluster, consisting of three genes (*CHD2, SCN10A*, and *TBC1D8*), was associated with cellular assembly and organization, nervous system development and function, and ion channels, as well as neurological disease, skeletal and muscular disorders, and behavioral disorders (Figure 4B). These data indicate that the candidate genes play important roles in LGS development.

**TABLE 3:** Gene ontology results of candidate genes of LGS or LGS-like epilepsy.

Gene symbol	Description	GO term	Function	Related pathway
FRRS1L	Ferric chelate reductase 1 like	GO:0005886	Plasma membrane	AMPA receptor biogenesis
IQSEC2	IQ motif and Sec7 domain 2	GO:0030036	Actin cytoskeleton organization	AMPA receptor regulation
SSPO	SCO-spondin	GO:0007155	Cell adhesion	Central nerve system formation
CHD2	Chromodomain helicase DNA binding protein 2	GO:0006351	Transcription	Chromatin remodeling
SLC25A39	Solute carrier family 25 member 39	GO:0006412	Translation	Heme biosynthesis
CACNA1A	Calcium voltage-gated channel subunit alpha1 A	GO:0000096	Sulfur amino acid metabolic process	Ion channel
SCN10A	Sodium voltage-gated channel alpha subunit 10	GO:0002027	Regulation of heart rate	Ion channel
MAGI1	Membrane associated guanylate kinase, WW and PDZ domain containing 1	GO:0006461	Protein complex assembly	Neurite outgrowth
SHANK3	SH3 and multiple ankyrin repeat domains 3	GO:0000165	MAPK cascade	Neurotransmission
SYN1	Synapsin I	GO:0007268	Chemical synaptic transmission	Neurotransmitter release cycle
SYN2	Synapsin II	GO:0007268	Chemical synaptic transmission	Neurotransmitter release cycle
DNAJC5	Dnaj heat shock protein family (Hsp40) member C5	GO:0006887	Exocytosis	Protein folding
NRG2	Neuregulin 2	GO:0000165	MAPK cascade	Regulates neurite outgrowth and neuron cell survival
TBC1D8	TBC1 domain family member 8	GO:0006886	Intracellular protein transport	Vesicle-mediated transport

*Abbreviation: GO, Gene ontology.*

### Neurite Alteration in *SLC25A39* and *TBC1D8* Knockdown Cell Lines

**FIGURE 5:**
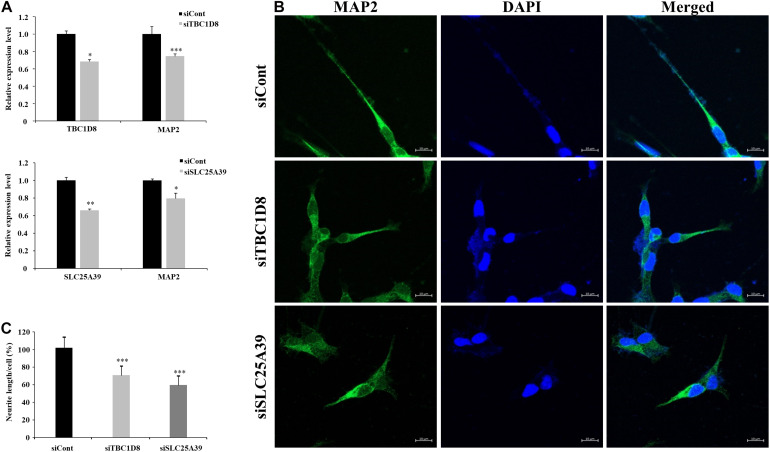
Neurite length analysis by knockdown of candidate genes in neuronal cells. Human SH-SY5Y neuroblastoma cells were transfected with siRNA for *TBC1D8* and *SLC25A39* on day 3 *in vitro* (DIV 3). *MAP2* was used as a neuronal marker. **(A)** Expression levels of *TBC1D8*, *SLC25A39*, and *MAP2* were analyzed by RT-PCR (*n* = 3). **(B)** After retinoic acid stimulation for 48 h, the cells were stained for *MAP2* (green) and nuclei (blue). Scale bars: 30 μm. **(C)** Neurite length was measured in more than 10 cells in three independent experiments. ^∗^*p* < 0.05, ^∗∗^*p* < 0.01, ^∗∗∗^*p* < 0.001, and Student’s *t*-test compared with the control group.

Among neuron-related genes, the direct neuronal functions of *SLC25A39* and *TBC1D8* have not been reported: *Shawn*, the *Drosophila* homolog of *SLC25A39* and *SLC25A40*, reportedly plays a role in neurotransmitter release (Slabbaert et al., 2016) and *TBC1D24*, a member of the protein family of *TBC1D8*, regulates neuronal migration (Riazuddin et al., 2017). To examine the effects of *SLC25A39* and *TBC1D8* on neuronal function, we depleted *SLC25A39* and *TBC1D8* from the SH-SY5Y neuroblastoma cell line and examined neurite outgrowth. siRNA treatment of target genes decreased *SLC25A39* and *TBC1D8* expression by approximately 40%. Under retinoic acid-induced neuronal differentiation conditions, the mRNA level of *MAP2* was significantly decreased in *SLC25A39* or *TBC1D8* knockdown cells (Figure 5A). Additionally, the neurite length was reduced by 30% in the *TBC1D8* knockdown group and by 40% in the *SLC25A39* knockdown group compared to in the control, indicating impaired neuronal development (Figures 5B,C). These results suggest that *SLC25A39* and *TBC1D8* are involved in the neurite extension of neuronal cells.

## Discussion

Genetic variations play an important role in the development of epileptic syndromes, including LGS without brain abnormalities, by altering neurotransmission or neuronal development (Falace et al., 2014; Lund et al., 2014; Fedele et al., 2018). In this study, we identified 14 candidate genes and genetic variations related to neuronal development or neurotransmission as biomarkers of LGS and LGS-like epilepsy with unknown causes. Of these, seven mutations were novel. In addition, two genes showed insufficient evidence for epilepsy or neuronal functions; however, we demonstrated that these genes affect neurite outgrowth in a human neuroblastoma cell line.

Pathogenicity evaluation of genetic variations in our candidate genes, with probability used as a risk factor for LGS and LGS-like epilepsy, revealed that several variations were pathogenic or likely pathogenic. In addition, variations reported in four genes, *IQSEC2*, *SYN1*, *SYN2*, and *FRRSIL*, were in a domain position similar to those in the candidate genes and classified as pathogenic or of uncertain significance according to ClinVar classification (Supplementary Table 2). Furthermore, the protein stability of the 10 candidate genes was altered by isoelectric point changes via side chain modifications of amino acids by genetic variations, implicating the effect of protein function (Supplementary Table 3). Collectively, these results indicate that our variations are associated with LGS and LGS-like epilepsy. However, genotype-phenotype data and gene-specific mutation rates for our candidate genes were not provided because these variants were rare.

Among the genes with 14 genetic variations, including seven novel mutations, *MAGI1*, *NRG2*, *SSPO*, *SLC25A39*, and *TBC1D8* were not associated with LGS or epilepsy. However, they were selected as candidate genes because of their roles in neuronal development. *MAGI1*, *NRG2*, and *SSPO* exert neuron-related functions. *MAGI1* plays a role in regulating neurite outgrowth (Ito et al., 2012). *SSPO* promotes neuronal survival and differentiation and is required during early brain development (Goncalves-Mendes et al., 2004; Vera et al., 2013). *NRG2* promotes neuronal survival and neurite extension (Nakano et al., 2016). *SLC25A39* resides in susceptibility loci for epilepsy with heterogeneous phenotypes but not as an epilepsy-related gene because its variant has not been identified in families with epilepsy (Siren et al., 2010). However, a variant of *SLC25A39* was identified in our LGS data, and depletion of *SLC25A39* decreased neurite outgrowth in human neuroblastoma cells. These data suggest a relationship between LGS and epilepsy with *SLC25A39*. These findings are also supported by previous studies in which mutations in *Shawn*, the *Drosophila* homolog of *SLC25A39* and *SLC25A40*, increase neurotransmitter release (Slabbaert et al., 2016). *TBC1D24*, a member of the same protein family as *TBC1D8*, regulates neuronal migration and its epilepsy-related mutation is in the Rab-GAP TBC domain, which is the same domain containing the mutation evaluated in our study (Falace et al., 2014; Banuelos et al., 2017). Alternatively, mutations in *TBC1D8* have been detected in patients with intellectual disability without an epilepsy phenotype (Riazuddin et al., 2017). However, our data revealed mutations of *TBC1D8* in Korean patients with LGS as well as decreased neurite outgrowth in human neuroblastoma cells by depletion of *TBC1D8*, suggesting that *TBC1D8* is a candidate gene for LGS or epilepsy. Epileptic seizure is defined as the transient occurrence of signs and/or symptoms because of abnormal excessive or synchronous neutral activity in the brain. Previous studies showed that epilepsy or LGS phenotypes may occur when neurotransmission and neuronal development in the brain are disrupted (Hermann et al., 2002; Staley, 2015; Fukata and Fukata, 2017; Falco-Walter et al., 2018; Jahngir et al., 2018). Collectively, these findings indicate that mutations in these five genes can trigger the LGS phenotype by disrupting neuronal development.

The results of network analysis revealed that the 11 genes *CACNA1A*, *DNAJC5*, *FRRS1L, IQSEC2*, *MAGI1*, *NRG2*, *SHANK3*, *SLC25A39*, *SSPO*, *SYN1*, and *SYN2* are directly or indirectly associated with *ERK1/2*, *AKT*, and *KRAS*, which play important roles in neuronal survival, neural stem cell proliferation, and neurotransmission by α-amino-3-hydroxy-5-methyl-4-isoxazolepropionic acid (AMPA) and N-methyl-d-aspartate receptors (Nateri et al., 2007; Subramaniam and Unsicker, 2010; Bender et al., 2015). Therefore, genetic variations in genes from this group can induce seizure phenotypes of LGS because of both disrupted neuronal development and neurotransmission via ion channels in the brain. Another group of genes, *CHD2*, *SCN10A*, and *TBC1D8*, was associated with *HGF* and *PTEN*; these two genes are related to synaptic plasticity, neuronal cell survival, and CNS development, as well as related to epilepsy (Bae et al., 2010; Tonges et al., 2011; Sperow et al., 2012; Knafo and Esteban, 2017; Godale and Danzer, 2018). These results indicate that variations within candidate genes can impair protein function, directly or indirectly affect neurotransmission or neuronal development pathways, and subsequently induce LGS or LGS-like epilepsy. Our results are in accordance with a previous study in which homozygous mutations in *STXBP1* were identified in two siblings diagnosed with LGS. The mutations resulted in impairment of protein stability leading to reduced synaptic transmission (Lammertse et al., 2020).

Children with LGS have a high percentage of severe injuries as well as other comorbidities, with a potential increased risk of death (Autry et al., 2010). These children were 14-fold more likely than those of the general population to die, indicating that the genetic variation causing LGS is difficult to detect in the next generation. This is likely because there was no LGS family with AD in our study. In addition, the two patients with *SYN2* and *MAGI1* variations in our datasets experienced infantile spasms. The variations may have been responsible for these effects and subsequently led to LGS, as 30% of LGS patients can gradually progress from infantile spasms (Cross et al., 2017).

In summary, we identified mutations in 14 genes as potential causative markers of both LGS and LGS-like epilepsy. Many of these candidate genes are generally pathogenic and are associated with neurotransmission or neuronal function. These results expand the spectrum of variations in LGS and LGS-like epilepsy and are crucial for understanding their biological mechanisms for patient-specific therapeutic development. Further animal model studies are required to determine whether these genetic variations can disrupt neuronal function and cause the LGS phenotype.

## Data Availability Statement

The dataset generated for this study was submitted to the Seqeuence Read Archive (SRA) and can be accessed by searching the BioProject ID PRJNA601231 on the NCBI website (https://www.ncbi.nlm.nih.gov/bioproject/PRJNA601231).

## Ethics Statement

The studies involving human participants were reviewed and approved by Institutional Review Board and Ethics Committe at the Chungnam National University Hospitals and the Korea Research Institute of Bioscience and Biotechnology (KRIBB). Written informed consent to participate in this study was provided by the participants’ legal guardian/next of kin. Written informed consent was obtained from the individual(s), and minor(s)’ legal guardian/next of kin, for the publication of any potentially identifiable images or data included in this article.

## Author Contributions

N-SK and J-WK conceived and designed the study. JY, M-HC, J-YY, and N-SK drafted the manuscript. JY performed breakpoints verification. JY, M-HC, J-YY, J-JL, and JL participated in the data analysis. SY, S-JJ, SJ, IB, DH, and BL reviewed and edited the manuscript and contributed to the discussions. SN, JK, HK, and JL participated in clinical data collection. N-SK supervised the study. All authors reviewed and approved this submission.

## Conflict of Interest

The authors declare that the research was conducted in the absence of any commercial or financial relationships that could be construed as a potential conflict of interest.

## Footnotes

1 https://www.internationalgenome.org/
