# Corrigendum: Characteristics of Genetic Variations Associated With Lennox-Gastaut Syndrome in Korean Families

**DOI:** 10.3389/fgene.2021.669107

**Published:** 2021-03-05

**Authors:** Jin Ok Yang, Min-Hyuk Choi, Ji-Yong Yoon, Jeong-Ju Lee, Sang Ook Nam, Soo Young Jun, Hyeok Hee Kwon, Sohyun Yun, Su-Jin Jeon, Iksu Byeon, Debasish Halder, Juhyun Kong, Byungwook Lee, Jeehun Lee, Joon-Won Kang, Nam-Soon Kim

**Affiliations:** ^1^Korea BioInformation Center, Korea Research Institute of Bioscience and Biotechnology, Daejeon, South Korea; ^2^Department of Bio and Brain Engineering, Korea Advanced Institute of Science and Technology, Daejeon, South Korea; ^3^Rare-Disease Research Center, Korea Research Institute of Bioscience and Biotechnology, Daejeon, South Korea; ^4^Department of Functional Genomics, Korea University of Science and Technology, Daejeon, South Korea; ^5^Department of Pediatrics, Pusan National University Children's Hospital, Pusan National University School of Medicine, Yangsan, South Korea; ^6^Department of Medical Science and Anatomy, Chungnam National University, Daejeon, South Korea; ^7^Department of Pediatrics, Samsung Medical Center, Sungkyunkwan University School of Medicine, Seoul, South Korea; ^8^Department of Pediatrics and Medical Science, Chungnam National University Hospital, College of Medicine, Chungnam National University, Daejeon, South Korea

**Keywords:** Lennox-Gastaut syndrome, epilepsy, whole-exome sequencing, genetic variation, Rare-diseases

In the published article, there were errors in affiliations for authors “Iksu Byeo” and “Byungwook Lee.” Instead of “Department of Bio and Brain Engineering, Korea Advanced Institute of Science and Technology, Daejeon, South Korea”, it should be “Korea BioInformation Center, Korea Research Institute of Bioscience and Biotechnology, Daejeon, South Korea” for both the authors.

The authors apologize for this error and state that this does not change the scientific conclusions of the article in any way. The original article has been updated.

