# Supplementary figures and images for: Characteristics of Genetic Variations Associated With Lennox-Gastaut Syndrome in Korean Families
Source: Front Genet. 2021 Jan 20;11:590924. doi: 10.3389/fgene.2020.590924 (PMC7874053; doi:10.3389/fgene.2020.590924)

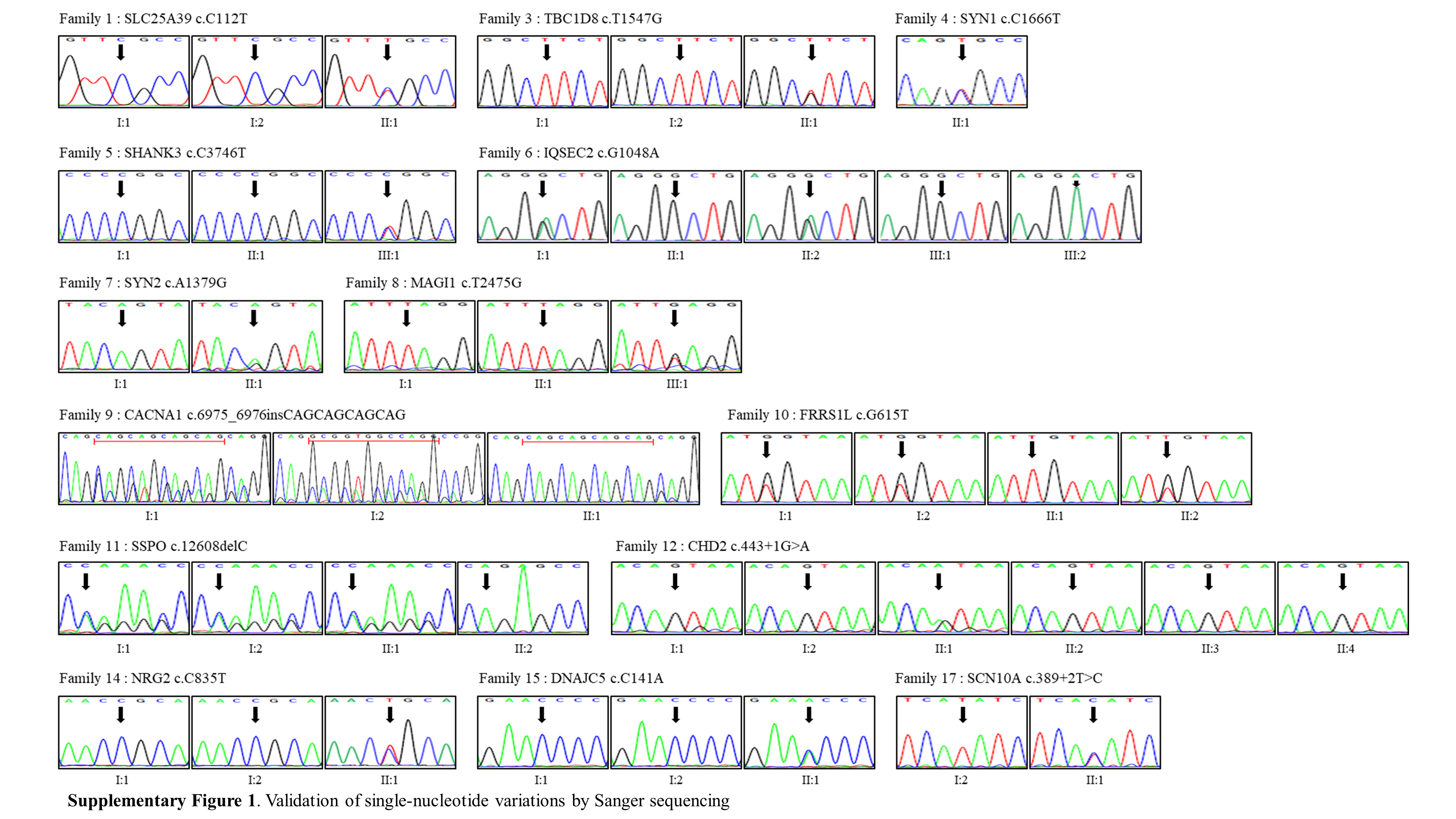

Supplement: Supplementary file 1 [file Image_1.tif]
