# Supplementary material for: Characteristics of Genetic Variations Associated With Lennox-Gastaut Syndrome in Korean Families
Source: Front Genet. 2021 Jan 20;11:590924. doi: 10.3389/fgene.2020.590924 (PMC7874053; doi:10.3389/fgene.2020.590924)
Supplement: Supplementary file 2 [file Table_1.DOCX]

**Supplementary Table 1.** Allele frequency and pathogenicity level of genetic variations in patients with LGS or LGS-like epilepsy

| Patient | Gene name | NT, AA change | Allele frequency | | ACMG/AMP | | |
| --- | --- | --- | --- | --- | --- | --- | --- |
|  |  |  | gnomAD | 1000 genome | Criteria | | Classification |
| Pathogenic or Likely pathogenic genetic variations | | | | | |  |  |
| 10 | ***FRRS1L*** | c.G615T , p.M205I | 0  (homozygotes) | NA | PVS1,PM1,PM2,  PM3, PP3 | | Pathogenic |
| 12 | ***CHD2*** | c.443+1G>A | NA | NA | PVS1,PS2,PM2,PP3 | | Pathogenic |
| 17 | ***SCN10A*** | c.389+2T>C | NA | NA | PVS1,PM2,PP3 | | Pathogenic |
| 4 | ***SYN1*** | c.C1666T, p.R556C | 0 | NA | PM1,PM2,PM6,PP3 | | Likely pathogenic |
| 6 | ***IQSEC2*** | c.G1048A, p.A350T | NA | NA | PM1,PM2,PM5,PP3 | | Likely pathogenic |
| 15 | ***DNAJC5*** | c.C141A, p.N47K | NA | NA | PS2,PM2,PP3 | | Likely pathogenic |
| Uncertain significance genetic variations | | | | | |  |  |
| 1 | ***SLC25A39*** | c.C112T, p.R38C | 0.000008032 | NA | PM2, PP3 | | Uncertain significance |
| 3 | ***TBC1D8*** | c.T1547G, p.L516R | NA | NA | PM2, PP3 | | Uncertain significance |
| 5 | ***SHANK3*** | c.C3746T, p.P1249L | 0.0001813 | NA | PM6, PP3 | | Uncertain significance |
| 7 | ***SYN2*** | c.A1379G, p.Q460R | 0.0001391 | 0.0002 | PM2,PM6, PP3 | | Uncertain significance |
| 8 | ***MAGI1*** | c.T2475G, p.F825L | NA | NA | PM2, PP3 | | Uncertain significance |
| 9 | ***CACNA1A*** | c.6975_6976insCAGCAGCAGCAG,  p.Q2325_A2326insQQQQ | NA | NA | PM3 | | Uncertain significance |
| 11 | ***SSPO*** | c.12608delC, p.Q4204Rfs*41 | 0.01506 (homozygotes) | NA | PM3, PM4 | | Uncertain significance |
| 14 | ***NRG2*** | c.C835T, p.R279C | 0.000007954 | NA | PM2, PP3 | | Uncertain significance |

Abbreviations: NT, Nucleotide; AA, Amino acid; NA, Not available

Allele frequency is from gnomAD and 1000 genome.
