# Supplementary material for: Characteristics of Genetic Variations Associated With Lennox-Gastaut Syndrome in Korean Families
Source: Front Genet. 2021 Jan 20;11:590924. doi: 10.3389/fgene.2020.590924 (PMC7874053; doi:10.3389/fgene.2020.590924)
Supplement: Supplementary file 3 [file Table_2.DOCX]

**Supplementary Table 2** Reported variations in a position similar to our variations

| Gene name | NT, AA change | rsID | ClinVar classification |
| --- | --- | --- | --- |
| *SLC25A39* | None | None | None |
| *TBC1D8* | None | None | None |
| *SYN1* | c.G1648A, p.A550T | rs397514680 | Pathogenic |
| *SHANK3* | None | None | None |
| *IQSEC2* | c.C1075T, p.R359C | rs267607188 | Pathogenic |
| *SYN2* | c.G1390C, p.D464H | rs751232089 | Uncertain significance |
| *MAGI1* | None | None | None |
| *CACNA1A* | None | None | None |
| *FRRS1L* | c.A607C, p.K203Q | None | Uncertain significance |
| *SSPO* | None | None | None |
| *CHD2* | None | None | None |
| *NRG2* | None | None | None |
| *DNAJC5* | None | None | None |
| *SCN10A* | None | None | None |

Abbreviations: NT, Nucleotide; AA, Amino acid; rsID, Reference SNP ID
