# Supplementary material for: Characteristics of Genetic Variations Associated With Lennox-Gastaut Syndrome in Korean Families
Source: Front Genet. 2021 Jan 20;11:590924. doi: 10.3389/fgene.2020.590924 (PMC7874053; doi:10.3389/fgene.2020.590924)
Supplement: Supplementary file 4 [file Table_3.DOCX]

**Supplementary Table 3** Protein stability changes by single-nucleotide variations

| **Protein** | **AA change** | **Side chain change** | **Properties change** | **PI change** | **I-Mutant 3.0** | |  |
| --- | --- | --- | --- | --- | --- | --- | --- |
|  |  |  |  |  | **DDG**  **(kcal/mol)** | **SVM2 Prediction** | **RI** |
| **SLC25A39** | R38C | Polar, positive → Polar, uncharged | Hydrophilic → Hydrophilic | 10.76 → 8.18 | -0.93 | Decrease | 4 |
| **TBC1D8** | L531R | Non-polar → Polar, positive | Hydrophobic → Hydrophilic | 5.98 → 10.76 | -1.70 | Decrease | 6 |
| **SYN1** | R556C | Polar, positive → Polar, uncharged | Hydrophilic → Hydrophilic | 10.76 → 8.18 | -0.37 | Increase | 1 |
| **SHANK3** | P1249L | Non-polar → Non-polar | Hydrophobic → Hydrophobic | 6.30 → 5.98 | -0.47 | Increase | 2 |
| **IQSEC2** | A350T | Non-polar → Polar, uncharged | Hydrophobic → Hydrophilic | 6.00 → 5.60 | -0.92 | Decrease | 8 |
| **SYN2** | Q460R | Polar, uncharged → Polar, positive | Hydrophilic → Hydrophilic | 5.65 → 10.76 | 0.19 | Increase | 6 |
| **MAGI1** | F853L | Non-polar → Non-polar | Hydrophobic → Hydrophobic | 5.48 → 5.98 | -0.91 | Decrease | 5 |
| **FRRS1L** | M205I | Non-polar → Non-polar | Hydrophobic → Hydrophobic | 5.74 → 6.02 | -0.77 | Decrease | 6 |
| **NRG2** | R279C | Polar, positive → Polar, uncharged | Hydrophilic → Hydrophilic | 10.76 → 8.18 | -1.26 | Decrease | 6 |
| **DNAJC5** | N47K | Polar, uncharged → Polar, positive | Hydrophilic → Hydrophilic | 5.41 → 9.74 | -0.14 | Decrease | 3 |

Abbreviations: AA, Amino acid; PI, Isoelectric point; DDG, Free energy change value; SVM, Support vector machine (DDG < -0.5: Decrease stability, -0.5 =< DDG =< 0.5: Weak effect, DDG > 0.5: Increase stability); RI: Reliability index (Range 0-9)
