# Supplementary material for: Characteristics of Genetic Variations Associated With Lennox-Gastaut Syndrome in Korean Families
Source: Front Genet. 2021 Jan 20;11:590924. doi: 10.3389/fgene.2020.590924 (PMC7874053; doi:10.3389/fgene.2020.590924)
Supplement: Supplementary file 5 [file Table_4.DOCX]

**Supplementary Methods**

**1.1 | Validation analysis**

**Sanger sequencing.** PCR was performed to verify the single-nucleotide variations selected by whole-exosome sequencing data analysis. The PCR products were bidirectionally sequenced by Sanger sequencing on an ABI3730XL DNA sequencer.

**Preparation of microarray library and genotyping.** CNV candidate samples were genotyped on three Illumina Infinium Omni2.5-8 BeadChip using the Infinium LCG Assay workflow (https://www.illumina.com/products/by-type/microarray-kits/infiniumomni25-8.html). A total of 400 ng of genomic DNA was extracted and amplified to generate a sufficient quantity of each DNA sample. The amplified DNA samples were fragmented and hybridized overnight on an Omni2.5-8 BeadChip. The loaded BeadChips were subjected to single-base extension and staining, followed by imaging on an iScan machine to obtain genotyping information. The genotyping data were exported from Illumina GenomeStudio to ped and map pairs, merged, and converted into VCF format using PLINK v1.980. Variants missing in more than 5% of the samples were removed.
